# The Effectiveness of an Emotional Intelligence Training Program on Emotional Intelligence and Multidimensional Self‐Efficacy: A Quasi‐Experimental Study

**DOI:** 10.1155/jonm/7930140

**Published:** 2026-10-04

**Authors:** Shaherah Yousef Andargeery, Enas Mohammed Lotfy

**Affiliations:** ^1^ Department of Nursing Management and Education College of Nursing Princess Nourah bint Abdulrahman University P.O. Box 84428 Riyadh 11671 Saudi Arabia pnu.edu.sa; ^2^ Public Health Nursing Department College of Nursing Northern Border University Arar Saudi Arabia nbu.edu.sa

**Keywords:** educational intervention, emotional intelligence, nursing students, quasi-experimental design, self-efficacy

## Abstract

**Aim:**

To evaluate the effectiveness of a structured emotional intelligence training program on emotional intelligence and multidimensional self‐efficacy among undergraduate nursing students.

**Background:**

Emotional intelligence is recognized as a core competency in nursing education, supporting emotional regulation, interpersonal effectiveness, and academic persistence in demanding clinical environments. Emotional intelligence development remains insufficiently embedded within many undergraduate nursing curricula.

**Methods:**

A quasi‐experimental two‐group pretest–posttest study design was conducted with 119 undergraduate nursing students in Saudi Arabia. Students were assigned to an intervention group (*n* = 62) or a control group (*n* = 57). The Schutte Self‐Report Emotional Intelligence Test and multidimensional self‐efficacy were used to measure the variables. Independent‐samples and paired‐samples *t*‐tests were used for between‐ and within‐group comparisons.

**Results:**

Postintervention, the intervention group demonstrated significantly higher emotional intelligence scores (*M* = 134.19, SD = 9.79) than the control group (*M* = 126.32, SD = 16.26), *t*(90.33) = 3.168, *p* = 0.002, *d* = 0.598. The intervention group also showed significant improvements across all five self‐efficacy dimensions (*d* = 0.299–1.219), whereas the control group showed minimal change. Within‐group analyses revealed large to very large pre–post improvements in the intervention group (*d* = 0.275–1.066), with no significant changes in the control group except academic self‐efficacy (*d* = 0.284, *p* = 0.036).

**Discussion:**

Emotional intelligence competencies can be developed through brief, structured educational interventions and that emotional intelligence enhancement is linked to gains in students’ self‐efficacy and professional readiness outcomes.

**Conclusion:**

Structured emotional intelligence training significantly enhances emotional intelligence and multidimensional self‐efficacy among undergraduate nursing students.

**Implications for Nursing:**

Integrating emotional intelligence training into undergraduate nursing curricula may strengthen students’ emotional preparedness and academic confidence.

**Implications for Nursing Policy:**

Nursing education policies should recognize emotional intelligence as a core professional competency and support its integration into curriculum standards.

**Implications for Nursing Management:**

Nursing education leaders and academic administrators should prioritize structured, competency‐based communication training and language support initiatives within nursing curricula to strengthen students’ humanistic practice abilities and improve future patient‐centered care outcomes.

## 1. Background

Emotional intelligence (EI) has gained increasing recognition as a critical competency in health professions education, particularly in nursing, where students and practitioners routinely operate in emotionally demanding, high‐pressure clinical environments [1]. EI is defined as the ability to recognize, understand, regulate, and express emotions both intrapersonally and interpersonally [2]. In nursing practice, EI underpins essential professional competencies, such as therapeutic communication, empathy, teamwork, ethical decision‐making, and clinical judgment [3–5]. Higher EI levels are associated with improved coping, resilience, and collaborative practice in clinical settings [6, 7].

Growing empirical evidence supports the integration of EI development into nursing education. Structured EI interventions have been shown to enhance students’ emotional awareness, regulation, stress management, and psychological well‐being [8–10], while also reducing burnout and improving interpersonal competencies [1, 2]. In clinical education contexts, EI development has been linked to improved communication skills and reduced anxiety during patient care encounters [12].

Self‐efficacy was defined as individuals’ beliefs in their capacity to execute tasks and manage challenges [13]. It represents another key psychological construct influencing academic and professional performance. Among nursing students, higher self‐efficacy is associated with stronger academic achievement, sustained engagement, perceived competence, and resilience [14, 15]. Elevated self‐efficacy also buffers stress and promotes coping [16], with students reporting greater educational satisfaction and commitment to professional excellence [17, 18].

A positive and theoretically meaningful relationship exists between EI and self‐efficacy. Nursing students with higher EI tend to demonstrate stronger self‐efficacy beliefs, which in turn support effective coping, academic engagement, and performance under pressure [19]. EI facilitates self‐regulatory processes including emotional awareness, adaptive emotion management, and motivation, which are foundational to self‐efficacy development. Emotionally intelligent students are better equipped to manage learning demands, collaborate effectively, and maintain intrinsic motivation through enhanced self‐efficacy [20, 21].

Despite this recognition, structured EI training remains insufficiently embedded within undergraduate nursing curricula, with emotional learning often addressed implicitly rather than through formally designed interventions [22]. This gap is concerning given the increasing complexity of healthcare environments and the heightened emotional demands placed on nursing students during clinical training. Furthermore, most prior studies have focused on overall EI outcomes or general self‐efficacy, with limited attention to how EI development translates into specific domains of student functioning [23]. The relationship between EI training and multidimensional self‐efficacy including cognitive and academic domains directly linked to learning processes remains underexplored. Many existing interventions are also time‐intensive, limiting their feasibility for integration into dense nursing curricula.

Although EI is frequently examined within educational contexts, its relevance extends beyond academic development and is increasingly recognized as a critical competency for nursing leadership and workforce effectiveness [24]. EI has been associated with effective communication, conflict management, resilience, teamwork, leadership performance, and employee retention [25]. Consequently, educational interventions that strengthen EI during undergraduate preparation may contribute to the development of a more adaptable, emotionally competent, and leadership‐ready nursing workforce [26]. From a nursing management perspective, understanding strategies that enhance these competencies early in professional development may support long‐term workforce sustainability and organizational effectiveness.

To address these gaps, this study aimed to evaluate the effectiveness of a structured, short‐duration EI training program on EI and multidimensional self‐efficacy among undergraduate nursing students.

*Primary Aim*: To examine the effect of the EI intervention on overall EI scores compared with a control group receiving usual academic instruction.

*Secondary Aims*: (1) To examine the effect of the EI intervention on five distinct dimensions of self‐efficacy (emotional, social, insistence, cognitive, and academic).

(2) To assess within‐group changes from pretest to posttest in EI and all self‐efficacy dimensions for both the intervention and control groups.

By adopting a multidimensional perspective and implementing a brief, scalable intervention within an authentic educational setting, this study extends existing literature by providing deeper insight into how EI development influences distinct domains of self‐efficacy and supports both emotional and academic readiness in nursing education.

The novelty of this study lies in three key areas: First, whereas many previous EI intervention studies have focused primarily on changes in overall EI or general self‐efficacy, this study examined five distinct dimensions of self‐efficacy, providing a more nuanced understanding of how EI development may influence emotional, social, cognitive, and academic functioning. Second, the intervention was intentionally designed as a brief, structured, and curriculum‐compatible program, enhancing its feasibility for integration into undergraduate nursing education. Third, evidence regarding EI interventions among nursing students in Saudi Arabia remains limited, and little is known about their influence on multidimensional self‐efficacy [27]. By addressing these gaps, the present study contributes new evidence regarding both the effectiveness and practical applicability of EI training in nursing education.

## 2. Methods

### 2.1. Study Design

This study employed a quasi‐experimental two‐group pretest–posttest design to evaluate the effectiveness of a structured EI training program on EI and multidimensional self‐efficacy among undergraduate nursing students. The study used pre‐existing academic sections as the units of group allocation. Fourth‐year nursing students were already enrolled in one of four academic sections through the College’s routine self‐registration process before the study commenced. To allocate the intervention, the researchers randomly selected two of the four existing sections to receive the EI training program, while the remaining two sections served as the comparison group and continued their usual academic instruction without receiving the EI training. Thus, students were not individually randomized to the intervention or comparison condition; rather, allocation occurred at the academic‐section level. This approach was adopted to facilitate implementation within the existing academic structure, minimize disruption to students’ scheduled coursework and clinical activities, and reduce the potential for contamination between students receiving and not receiving the intervention.

### 2.2. Setting and Participants

The study was conducted at a public College of Nursing in Riyadh, Saudi Arabia, during the second semester of the 2024 academic year. The target population consisted of fourth‐year undergraduate students enrolled in the Bachelor of Science in Nursing (BSN) program. At the time of the study, fourth‐year students were organized into four academic sections. Students had self‐registered into these sections through the College’s routine course‐registration process before recruitment for the study.

All students within the selected sections were invited to participate in the study and were informed that participation was voluntary. Students who met the eligibility criteria and provided written informed consent were included in the study. Two of the four existing academic sections were randomly selected by the researchers to constitute the intervention group, while the other two sections constituted the control group. Students therefore did not select whether their section would receive the intervention; rather, their intervention or comparison status was determined by the section in which they had previously enrolled and the subsequent random selection of sections by the researchers.

### 2.3. Sample Size Determination

A priori power analysis was conducted using G∗Power 3.1 to determine the minimum required sample size. Assuming a medium effect size (Cohen’s *d* = 0.50), a significance level of *α* = 0.05, and statistical power of 0.95, the analysis indicated that a minimum of 55 participants was required. A total of 119 students met the eligibility criteria and completed both pretest and posttest assessments, with 62 students in the intervention group and 57 students in the control group.

### 2.4. Intervention

The intervention consisted of a structured EI training program designed to enhance nursing students’ emotional competencies and multidimensional self‐efficacy relevant to academic learning and clinical practice. The program was developed based on established EI frameworks and evidence‐based educational strategies used in health professions education.

The EI training program was delivered over a period of four consecutive weeks, with two sessions per week, resulting in a total of eight sessions. Each session lasted approximately 30 min. Sessions were scheduled during designated academic break periods within the College of Nursing to minimize disruption to students’ regular coursework and clinical training.

The intervention addressed five core domains of EI that are considered essential for nursing education and professional practice which are self‐awareness, self‐regulation, motivation, empathy, and social skills [4, 28]. Each session was structured to introduce EI concepts, encourage reflection, and promote practical application of EI skills in real‐life nursing scenarios.

A variety of learner‐centered and interactive instructional strategies were employed to facilitate active engagement and experiential learning. These included brief interactive lectures to introduce key EI concepts, small‐group discussions to encourage peer interaction and shared reflection, and reflective exercises to enhance emotional awareness and self‐monitoring. These also included role‐playing scenarios based on academic and clinical situations to practice emotional regulation, empathy, and communication skills, and guided self‐assessment activities to support insight into personal emotional strengths and areas for development. These strategies were selected to promote deeper learning and to support the transfer of EI skills to both academic tasks and clinical practice.

The EI training program was delivered using a standardized session format across all intervention groups to ensure consistency in content and instructional approach. Session objectives, learning activities, and discussion prompts were predefined and followed systematically throughout the intervention period. Attendance was monitored for each session to ensure adequate exposure to the intervention content. Participants who attended the majority of sessions were considered to have received sufficient intervention dosage. The standardized delivery and consistent facilitation helped minimize variability in participant experience and strengthened internal validity.

Participants in the control group continued with their usual academic curriculum and did not receive any EI training during the study period. No additional educational or psychological interventions related to EI or self‐efficacy were provided to the control group. To reduce contamination, intervention content and materials were not shared with control group participants during the study period.

To enhance reproducibility, each session followed a structured format consisting of (1) brief conceptual input, (2) guided discussion, (3) experiential activity, and (4) reflective debriefing. Examples of session content and activities include the following: (1) completion of guided self‐reflection exercises by the participants to identify personal emotional triggers encountered in academic or clinical situations. For example, participants were asked to describe a recent stressful interaction and analyze their emotional responses and underlying thoughts. (2) Role‐playing scenarios were used to practice managing emotional reactions. For instance, students simulated a high‐pressure clinical situation involving patient distress and practiced techniques, such as pause‐and‐reframe and controlled breathing. (3) Goal‐setting exercises were conducted in which students identified short‐term academic and clinical goals and discussed emotional barriers to achieving them. (4) Perspective‐taking activities were implemented, where students analyzed patient scenarios and discussed appropriate emotional and communicative responses. (5) Small‐group discussions and communication exercises focused on assertiveness, active listening, and constructive feedback in peer and clinical interactions. Each session incorporated real‐life nursing‐related scenarios to facilitate transfer of learning to clinical practice.

To facilitate reproducibility, detailed session objectives, instructional activities, discussion prompts, and implementation procedures were documented in an intervention manual developed for this study. A summary of the intervention content and session structure is provided within the manuscript, and the full intervention materials are available from the corresponding author upon reasonable request.

### 2.5. Intervention Process and Allocation Procedure

The four existing fourth‐year academic sections were identified before commencement of the intervention. Because students had already self‐registered into their respective sections as part of the routine academic registration process, individual reassignment of students was not undertaken. To determine which sections would receive the EI training, the researchers randomly selected two of the four sections using a random selection procedure. The two selected sections were designated as the intervention group, and the remaining two sections were designated as the comparison group.

Students within the intervention sections who met the eligibility criteria and consented to participate received the eight‐session EI training program. Students in the two comparison sections continued to receive the usual academic curriculum and did not receive the EI training during the study period. This section‐level allocation was intended to preserve the existing academic organization and reduce the possibility that students in the intervention and comparison conditions would interact during the intervention and share intervention materials or activities.

Because the intervention was delivered to intact academic sections rather than to individually randomized students, the study was considered quasi‐experimental. Although the academic sections were randomly selected for the intervention condition, individual students were not randomly allocated to treatment conditions because their section membership had been established through the routine self‐registration process before the study. Participation in the research itself remained voluntary, and students could decline participation without academic consequences.

The intervention was delivered by one instructor with a background in nursing education and prior experience in teaching communication and professional skills. The facilitator held a PhD in Nursing. Before implementation, the facilitator underwent a structured orientation session conducted by the research team, which included reviewing the intervention manual and session objectives, standardization of instructional strategies and delivery approach, and training on facilitating reflective discussions and experiential learning activities. This preparation ensured consistency in delivery across sessions and minimized variability related to facilitator differences.

Several strategies were implemented to ensure intervention fidelity, which include a standardized intervention manual outlining session objectives, content, activities, and discussion prompts that were used across all sessions. Also, a session checklist was completed by the facilitator after each session to confirm that all planned components were delivered, and periodic informal observations were conducted by a member of the research team to ensure adherence to the protocol. These procedures helped maintain consistency in content delivery and ensured that participants received a comparable intervention experience.

Participant adherence to the intervention was excellent. All 62 students assigned to the intervention group attended all eight training sessions. Consequently, the attendance rate was 100%, and no missed sessions were recorded. The absence of attrition and complete attendance across all intervention sessions provided consistent exposure to the training content and further strengthened intervention fidelity.

### 2.6. Blinding and Mitigation of Detection Bias

Due to the nature of the educational intervention, blinding of participants and facilitators was not feasible. However, to minimize detection bias, outcome assessment was conducted using standardized self‐report instruments administered under consistent conditions for both groups. All questionnaires were coded anonymously, and data were de‐identified before analysis. A single‐blind approach was partially implemented at the data analysis stage. Group labels were masked (coded as Group A and Group B) during statistical analysis to reduce potential analytical bias.

### 2.7. Measurements

#### 2.7.1. Demographic Characteristics

Participants completed a demographic questionnaire that collected information on age, marital status, and cumulative grade point average (GPA). These variables were used to describe the sample and assess baseline comparability between the intervention and control groups.

#### 2.7.2. EI

EI was assessed using the Schutte Self‐Report Emotional Intelligence Test (SSEIT) developed by Schutte et al. [29]. The SSEIT is a widely used instrument based on the EI framework proposed by Salovey and Mayer [30] and O’Connor et al. [31]. The scale consists of 33 items rated on a five‐point Likert scale ranging from 1 (*strongly disagree*) to 5 (*strongly agree*). Three negatively worded items (items 5, 28, and 33) were reverse‐coded before analysis. Total scores range from 33 to 165, with higher scores indicating greater EI. The SSEIT has demonstrated strong psychometric properties in previous studies involving health professions students [32]. In this study, internal consistency reliability was excellent, with a Cronbach’s alpha coefficient of 0.938.

#### 2.7.3. Self‐Efficacy

Multidimensional self‐efficacy was measured using the Multidimensional Perceived Self‐Efficacy Scale, originally developed by Alwan [33] and later adapted for nursing students by Ibrahim et al. (2020). The scale consists of 39 items distributed across five dimensions: emotional self‐efficacy (8 items), social self‐efficacy (8 items), insistence and persistence self‐efficacy (8 items), cognitive self‐efficacy (8 items), and academic self‐efficacy (7 items). Items are rated on a five‐point Likert scale ranging from 1 (*never*) to 5 (*always*). Subscale scores were calculated by summing item responses within each dimension, with higher scores indicating greater perceived self‐efficacy.

The scale has demonstrated good reliability in an Arabic nursing context [34]. The instrument was culturally adapted and validated for use within the study context, supporting its construct validity and contextual relevance.

### 2.8. Data Collection Procedure

Data were collected using structured, self‐administered questionnaires at two time points: before the intervention (pretest) and immediately after the completion of the intervention (posttest). Before data collection, participants were informed of the study objectives, procedures, and the voluntary nature of participation. Pretest data were collected during the first week of the study for both groups under standardized classroom conditions. Posttest data were collected following the 4‐week intervention period, with both groups completing the assessment during the same timeframe to reduce potential time‐related bias. All questionnaires were completed in paper‐based format and coded anonymously to ensure confidentiality while allowing accurate matching of pretest and posttest responses.

### 2.9. Data Analysis

Data were analyzed using IBM SPSS Statistics Version 28. Descriptive statistics, including frequencies, percentages, means, and standard deviations, were used to summarize demographic characteristics and study variables. Data completeness was assessed before analysis. All participants who completed the baseline assessment also completed the postintervention assessment and were included in the final analyses. Therefore, no missing data were present, and no imputation procedures were required. In addition to reporting *p*‐values and effect sizes (Cohen’s d), 95% confidence intervals were calculated for the principal outcome estimates to provide information regarding the precision of the observed effects.

To enhance analytical transparency and reproducibility, the statistical procedures, assumptions, effect size calculations, confidence interval estimation methods, and data management procedures are described in detail. Independent‐samples *t*‐tests were conducted to compare mean scores of EI and self‐efficacy dimensions between the intervention and control groups at pretest and posttest. Paired‐samples *t*‐tests were used to examine within‐group changes from pretest to posttest for both groups. Statistical significance was set at *p* < 0.05. In addition to statistical significance testing, standardized effect sizes were calculated to assess the magnitude of observed differences. Cohen’s *d* was computed for both independent‐samples *t*‐tests (between‐group comparisons) and paired‐samples *t*‐tests (within‐group pre–post changes). For between‐group comparisons, Cohen’s *d* was calculated using pooled standard deviations, while for within‐group comparisons, it was calculated using the mean difference divided by the standard deviation of the differences. Effect sizes were interpreted according to conventional benchmarks, where 0.20 indicates a small effect, 0.50 a medium effect, and 0.80 or higher a large effect. The study employed a quasi‐experimental design, and analyses were conducted at the individual participant level using established procedures for pretest–posttest group comparisons.

### 2.10. Ethical Considerations

Ethical approval for the study was obtained from the Institutional Review Board of Princess Nourah bint Abdulrahman University, Riyadh, Saudi Arabia (Approval No. 23‐1043). All procedures were conducted in accordance with the Declaration of Helsinki and institutional research guidelines. Participants received detailed information about the study and provided written informed consent before participation. Participation was voluntary, and students were informed of their right to withdraw at any time without academic or personal consequences. Confidentiality and anonymity were strictly maintained, and all data were reported in aggregate form. As a noncoercive token of appreciation, participants in the intervention group received a certificate of attendance upon completion of the training program.

## 3. Results

### 3.1. Participant Characteristics

A total of 119 undergraduate nursing students participated in the study, with 62 students (52.1%) in the intervention group and 57 students (47.9%) in the control group. Demographic characteristics of the participants are summarized in Table 1. The majority of participants in both groups were aged 21 years or older, with comparable age distributions across groups. All students in the intervention group were single, while the control group included a small proportion of married students. Academic performance, as measured by cumulative GPA, was similar between groups, with most students reporting GPAs in the 3.75–4.49 range. Overall, the intervention and control groups demonstrated comparable demographic profiles at baseline.

**TABLE 1:** Demographic characteristics of the participants.

Demographics	*N* (%)	*N* (%)
	Intervention 62 (52.10%)	Control 57 (47.90%)
*Age*		
18–20	3 (2.52)	3 (2.52)
≥ 21	59 (49.58)	54 (45.38)

*Marital status*		
Single	62 (52.10)	50 (42.02)
Married	0	7 (5.88)

*GPA*		
4.50–5	23 (19.33)	23 (19.33)
3.75–4.49	32 (26.89)	26 (21.85)
2.75–3.74	7 (5.88)	8 (6.72)

Statistical comparisons of baseline demographic characteristics were conducted to assess group equivalence before the intervention. No statistically significant differences were observed between the intervention and control groups regarding age, marital status, or cumulative GPA (*p* > 0.05 for all comparisons). Furthermore, baseline comparisons of EI and all multidimensional self‐efficacy scores revealed no statistically significant between‐group differences (Table 2). These findings indicate that the intervention and control groups were comparable before implementation of the EI training program.

**TABLE 2:** Comparison between the mean scores of the intervention and control groups of emotional intelligence and self‐efficacy dimensions.

Variables	Pretest						Posttest					
	Intervention M (SD)	Control M (SD)	*t*‐value	*p* value	95% CI	ES	Intervention M (SD)	Control M (SD)	*t*‐value	*p* value	95% CI	ES
Emotional intelligence	123.47 (16.30)	126.75 (17.29)	−1.067	0.288	[−9.38, 2.81]	0.197	134.19 (9.79)	126.32 (16.26)	3.168	0.002	[2.94, 12.82]	0.598
SE‐Emotional dimension	22.90 (4.69)	21.23 (5.78)	1.741	0.087	[−0.23, 3.58]	0.322	25.16 (5.90)	22.54 (6.30)	2.340	0.021	[0.40, 4.83]	0.433
SE‐Social dimension	19.45 (4.33)	19.33 (4.39)	0.148	0.883	[−1.47, 1.70]	0.027	22.92 (6.47)	19.93 (5.23)	2.757	0.007	[0.84, 5.14]	0.510
SE‐Insistence dimension	20.87 (5.03)	19.07 (4.93)	1.968	0.051	[−0.01, 3.61]	0.364	23.89 (9.77)	18.54 (5.21)	3.676	< 0.001	[2.46, 8.22]	0.680
SE‐Cognitive dimension	20.73 (5.52)	19.70 (4.98)	1.059	0.292	[−0.89, 2.94]	0.196	25.18 (4.77)	21.02 (4.93)	4.678	< 0.001	[2.40, 5.92]	0.865
SE‐Academic dimension	17.24 (4.34)	16.82 (4.82)	0.497	0.620	[−1.25, 2.08]	0.092	24.06 (4.68)	18.67 (4.22)	6.591	< 0.001	[3.77, 7.02]	1.219

*Note:* ES: effect size using Cohen’s d.

### 3.2. Between‐Group Comparisons of EI and Self‐Efficacy

Independent‐samples *t*‐tests were conducted to compare EI and self‐efficacy scores between the intervention and control groups at pretest and posttest (Table 2). At pretest, no statistically significant differences were observed between the intervention and control groups in EI or any of the five self‐efficacy dimensions. Specifically, the pretest comparison was nonsignificant for EI (*p* = 0.288), emotional self‐efficacy (*p* = .087), social self‐efficacy (*p* = 0.883), insistence self‐efficacy (*p* = 0.051), cognitive self‐efficacy (*p* = 0.292), and academic self‐efficacy (*p* = 0.620). The corresponding effect sizes were small or negligible (*d* = 0.027–0.364), supporting baseline comparability between the two groups before the intervention.

At posttest, the intervention group demonstrated significantly higher EI scores (*M* = 134.19, SD = 9.79) than the control group (*M* = 126.32, SD = 16.26), (*t* = 3.168, *p* = 0.002, *d* = 0.598). The intervention group also demonstrated significantly higher scores across all five self‐efficacy dimensions at posttest. Emotional self‐efficacy was higher in the intervention group (*M* = 25.16, SD = 5.90) than in the control group (*M* = 22.54, SD = 6.30), (*t* = 2.340, *p* = 0.021, *d* = 0.433). Social self‐efficacy was also higher in the intervention group (*M* = 22.92, SD = 6.47) than in the control group (*M* = 19.93, SD = 5.23), (*t* = 2.757, *p* = 0.007, *d* = 0.510). For insistence self‐efficacy, the intervention group (*M* = 23.89, SD = 9.77) scored significantly higher than the control group (*M* = 18.54, SD = 5.21) (*t* = 3.676, *p* < 0.001, *d* = 0.680). Cognitive self‐efficacy was significantly higher in the intervention group (*M* = 25.18, SD = 4.77) than in the control group (*M* = 21.02, SD = 4.93) (*t* = 4.678, *p* < 0.001, *d* = 0.865). Finally, academic self‐efficacy was substantially higher in the intervention group (*M* = 24.06, SD = 4.68) than in the control group (*M* = 18.67, SD = 4.22) (*t* = 6.591, *p* < 0.001, *d* = 1.219).

The largest effects were observed for academic and cognitive self‐efficacy, followed by EI and insistence self‐efficacy, suggesting that the intervention had a meaningful educational impact beyond statistical significance.

### 3.3. Within‐Group Changes in EI and Self‐Efficacy

Paired‐samples *t*‐tests were performed to examine pretest–posttest changes within each group (Table 3). In the intervention group, statistically significant improvements from pretest to posttest were observed on EI and all self‐efficacy dimensions. EI increased significantly (*t* = −4.202, *p* < 0.001, *d* = 0.534, medium effect size). For self‐efficacy, the emotional dimension showed a significant increase (*t* = −2.351, *p* = 0.022, *d* = 0.299) (small effect), while the social dimension demonstrated a medium effect (*t* = −3.608, *p* = 0.001, *d* = 0.458). The insistence dimension also improved significantly (*t* = −2.165, *p* = 0.034, *d* = 0.275) (small effect). The cognitive dimension showed a medium‐to‐large effect (*t* = −4.872, *p* < 0.001, *d* = 0.619), and the academic dimension yielded a very large effect (*t* = −8.390, *p* < 0.001, *d* = 1.066).

**TABLE 3:** Paired‐sample *t*‐test results comparing pretest and posttest scores within intervention and control groups for emotional intelligence and self‐efficacy dimensions.

Variables	Control					Intervention				
	M(SD)	*t*‐value	*p* value	95% CI	ES	M(SD)	*t*‐value	*p* value	95% CI	ES
Emotional intelligence pretest–posttest	0.437 (25.09)	0.132	0.895	[−6.22, 7.10]	0.017	−10.726 (20.10)	−4.202	< 0.001	[−15.83, −5.62]	0.534
SE‐Emotional dimension pretest–posttest	−1.312 (8.17)	−1.217	0.229	[−3.48, 0.85]	0.161	−2.258 (7.56)	−2.351	0.022	[−4.18, −0.34]	0.299
SE‐Social dimension pretest–posttest	−0.596 (6.18)	−0.729	0.469	[−2.24, 1.05]	0.097	−3.468 (7.57)	−3.608	0.001	[−5.39, −1.55]	0.458
SE‐Insistence dimension pretest–posttest	0.526 (6.68)	0.586	0.560	[−1.27, 2.35]	0.078	−3.016 (10.97)	−2.165	0.034	[−5.80, −0.23]	0.275
SE‐Cognitive dimension pretest–posttest	−1.316 (6.02)	−1.651	0.104	[−2.91, 0.28]	0.219	−4.452 (7.20)	−4.872	< 0.001	[−6.28, −2.62]	0.619
SE‐Academic dimension pretest–posttest	−1.842 (6.48)	−2.146	0.036	[−3.56, −0.12]	0.284	−6.823 (6.40)	−8.390	< 0.001	[−8.45, −5.20]	1.066

In contrast, the control group showed no statistically significant changes from pretest to posttest on EI or on most self‐efficacy dimensions. EI did not change significantly (*t* = 0.132, *p* = 0.895, *d* = 0.017). Similarly, no significant improvements were found for the emotional dimension (*t* = −1.217, *p* = 0.229, *d* = 0.161), social dimension (*t* = −0.729, *p* = 0.469, *d* = 0.097), insistence dimension (*t* = 0.586, *p* = 0.560, *d* = 0.078), or cognitive dimension (*t* = −1.651, *p* = 0.104, *d* = 0.219). The only exception was the academic dimension, which showed a small but statistically significant increase (*t* = −2.146, *p* = 0.036, *d* = 0.284). The findings suggest that the EI training program was associated with improvements in EI and multidimensional self‐efficacy among participants in the intervention group.

## 4. Discussion

This study evaluated a structured, brief EI training program among undergraduate nursing students using a two‐group pretest–posttest quasi‐experimental design. The findings demonstrated significant improvements in overall EI and all five dimensions of self‐efficacy including emotional, social, insistence, cognitive, and academic, in the intervention group. Importantly, the intervention and control groups did not differ significantly at pretest on EI or any self‐efficacy dimension, supporting baseline comparability. At posttest, the intervention group had significantly higher scores than the control group across all measured outcomes, with between‐group effect sizes ranging from small to medium for emotional self‐efficacy to very large for academic self‐efficacy. Within‐group analyses further demonstrated significant pretest‐to‐posttest improvements in the intervention group for EI and all five self‐efficacy dimensions, whereas the control group showed no significant change in EI or four of the five self‐efficacy dimensions. Taken together, this pattern of baseline equivalence, intervention group improvement, and posttest between‐group differences provides evidence that participation in the structured EI training was associated with immediate improvements in EI and multidimensional self‐efficacy during the study period.

The significant improvement in EI observed in the intervention group is consistent with previous intervention research demonstrating that EI‐related competencies can be developed through structured educational programs [35]. In this study, the intervention group increased from a pretest mean of 123.47 to a posttest mean of 134.19, with a medium within‐group effect size (*d* = 0.534) and a medium between‐group posttest effect size (*d* = 0.598). These findings are comparable with recent intervention evidence among health professions students. For example, Dugué et al. [8] demonstrated that EI can be enhanced through training programs among health students, although the control group demonstrated less substantial improvements [36]. Similarly, a randomized controlled trial among nursing students demonstrated the potential of a structured EI program to improve EI among nursing students [37]. The consistency between these findings and the present results strengthens the interpretation that EI‐related competencies may be responsive to structured educational interventions rather than remaining entirely unchanged during undergraduate education.

These findings are also relevant to the Saudi nursing education context. A study of nursing students engaged in clinical practice in Saudi Arabia examined EI, self‐efficacy, and perceived stress, identifying EI and self‐efficacy as related psychological competencies among nursing students [19]. Although their study was cross‐sectional and therefore did not evaluate an intervention, its findings provide contextual evidence supporting the relevance of both constructs among Saudi nursing students. This study extends this evidence by using a pretest–posttest comparison and demonstrating that a structured educational intervention was associated with improvement in both constructs.

The improvement in EI may be related to the structure and content of the intervention. The program incorporated self‐reflection, guided discussion, role‐playing, perspective‐taking, and emotional regulation activities, thereby providing students with repeated opportunities to consider and practice emotional and interpersonal responses. Previous EI intervention research has similarly used combinations of theoretical instruction and experiential activities rather than relying solely on didactic teaching. For example, Dugué et al. [8] used a combination of theoretical and practical activities and reported improvements across several EI‐related domains. However, this study did not evaluate the independent contribution of each intervention component. Therefore, the observed improvement cannot be attributed to any specific activity. It is more appropriate to interpret the findings as evidence associated with participation in the overall structured EI program.

The magnitude of the EI effect observed in this study is also important. The between‐group effect was moderate (*d* = 0.598), rather than extremely large, suggesting a meaningful but not disproportionate intervention effect. This is broadly compatible with the heterogeneous EI intervention literature, in which the magnitude of improvement varies according to intervention duration, content, educational approach, participant characteristics, and outcome measurement [38]. Differences across studies should therefore not necessarily be interpreted as contradictory findings. Instead, variation may reflect differences in how EI is conceptualized and measured and in the intensity and delivery of the intervention. The present findings add to this literature by showing that a relatively brief program delivered within an undergraduate nursing context can produce measurable improvement in overall EI.

A particularly important finding was the significant improvement across all five dimensions of self‐efficacy. At posttest, the intervention group scored significantly higher than the control group in emotional self‐efficacy (*d* = 0.433), social self‐efficacy (*d* = 0.510), insistence self‐efficacy (*d* = 0.680), cognitive self‐efficacy (*d* = 0.865), and academic self‐efficacy (*d* = 1.219). The within‐group analysis showed the same overall pattern, with significant pretest‐to‐posttest improvement in all five dimensions. These results suggest that the observed association between EI training and self‐efficacy was not restricted to emotional or interpersonal functioning but extended to domains directly relevant to students’ learning and academic functioning.

The findings concerning emotional and social self‐efficacy are consistent with evidence linking EI and self‐efficacy among nursing students. Shubayr and Dailah [19] specifically examined the relationship between EI and self‐efficacy among nursing students in clinical practice in Saudi Arabia, highlighting the relevance of these constructs to nursing education and clinical preparation. These findings extend this relationship from an observational association to an intervention context by demonstrating significant improvement in emotional and social self‐efficacy following structured EI training. Nevertheless, because this study did not directly test whether changes in EI statistically mediated changes in self‐efficacy, the results should not be interpreted as demonstrating a mediation pathway.

The improvement in emotional self‐efficacy may be consistent with the focus of the intervention on emotional awareness and regulation. Students were provided with opportunities to identify emotional responses, reflect on emotional experiences, and practice strategies for managing emotions. Such activities are conceptually relevant to students’ perceptions of their ability to manage emotional demands. However, emotional regulation was not measured as a mediator in this study. Therefore, the improvement in emotional self‐efficacy should be interpreted as an observed outcome of the intervention rather than evidence that improved emotion regulation was the mechanism responsible for the change.

Similarly, the significant improvement in social self‐efficacy is compatible with the interpersonal nature of several intervention activities, including group discussion, role‐playing, perspective‐taking, and communication exercises. These activities provided opportunities for students to practice interpersonal responses in a structured learning environment. Previous EI intervention studies have reported improvements in social and interpersonal emotional competencies following structured training, providing support for the relevance of experiential and interactive approaches to EI development [39, 40]. Nevertheless, because this study did not separately measure changes in communication competence or interpersonal behavior, the observed improvement in social self‐efficacy cannot be attributed specifically to enhancements in those skills.

The most pronounced findings were observed for cognitive and academic self‐efficacy. Cognitive self‐efficacy showed a large between‐group effect (*d* = 0.865) and a medium‐to‐large within‐group effect (*d* = 0.619), whereas academic self‐efficacy showed a very large between‐group effect (*d* = 1.219) and a very large within‐group effect (*d* = 1.066). These findings are particularly noteworthy because they suggest that the intervention was associated not only with emotional and interpersonal outcomes but also with students’ perceptions of their learning‐related capabilities.

The larger effects observed in cognitive and academic self‐efficacy may be interpreted in light of the relationship between emotional functioning and students’ ability to manage demanding learning experiences. Previous literature has linked EI with academic functioning and self‐regulatory processes, suggesting that emotional competencies may be relevant to students’ ability to manage academic demands [41]. These findings are consistent with this broader literature, but they should not be interpreted as demonstrating that the intervention directly changed specific cognitive processes, such as attention, motivation, goal‐setting, or anxiety. These variables were not measured in this study.

The particularly large effect for academic self‐efficacy also warrants cautious interpretation. The intervention occurred during students’ ongoing academic experiences, and academic self‐efficacy may be influenced by multiple concurrent experiences. Although the intervention group demonstrated a substantially larger improvement than the control group, the present design cannot establish which specific elements of the intervention contributed to this large effect. It is therefore more appropriate to describe academic self‐efficacy as the outcome showing the strongest observed response to the intervention rather than to infer a specific psychological mechanism.

An additional consideration is that the control group demonstrated a small but statistically significant increase in academic self‐efficacy (*d* = 0.284), whereas no significant changes were observed for EI or the other self‐efficacy dimensions. This finding indicates that some change in academic self‐efficacy occurred during the study period independently of the EI intervention. Because the study did not measure students’ concurrent academic experiences, faculty support, peer learning, or other contextual influences, the reason for this small increase cannot be established. It would therefore be inappropriate to attribute the control group change to a particular mechanism. Importantly, the magnitude of the change was substantially smaller than that observed in the intervention group (*d* = 1.066), and the intervention group also demonstrated significant improvement across all other measured outcomes. The control group finding therefore does not negate the overall pattern favoring the intervention, but it reinforces the importance of avoiding overly simplistic causal interpretations.

Overall, these findings are consistent with a growing body of literature supporting structured EI education among health professions and nursing students. Recent evidence indicates that EI can be improved through educational interventions, although the magnitude and consistency of effects vary across studies [42]. Dugué et al. [8], for example, found significant improvements in several EI‐related competencies following an intervention among health students. A more direct study evaluated an EI program using a randomized controlled design among nursing students, providing further evidence that EI‐focused education can produce measurable changes in nursing students [37].

This study differs from these studies in several respects. First, it examined not only overall EI but also five distinct dimensions of self‐efficacy. Second, the intervention was brief, consisting of eight sessions of 30 min over 4 weeks. Third, the study was conducted among undergraduate nursing students in Saudi Arabia, providing evidence from a cultural and educational context that remains relatively underrepresented in EI intervention research [2]. The differences in intervention duration, content, educational setting, outcome measures, and participant characteristics should be considered when comparing effect sizes across studies. These methodological differences may explain why the magnitude of effects is not identical across studies.

These findings should therefore be viewed as complementary to rather than identical with previous intervention evidence. The convergence of findings across different health professions education settings supports the potential value of structured EI training, while differences in effect magnitude emphasize the need for further research to determine which intervention characteristics are most important for producing sustained improvements.

### 4.1. Theoretical Interpretation Through Social Cognitive Theory

The improvements in self‐efficacy can be interpreted within Bandura’s social cognitive theory, which proposes that self‐efficacy beliefs are influenced by enactive mastery experiences, vicarious experiences, verbal persuasion, and physiological and affective states [13]. Several features of the intervention were theoretically compatible with these sources of self‐efficacy. Role‐playing and experiential exercises provided opportunities for students to practice emotional and interpersonal responses; group activities provided opportunities to observe peers; guided discussions and facilitator feedback provided social reinforcement; and emotional awareness and regulation activities addressed students’ responses to affective experiences.

These theoretical links provide a coherent framework for interpreting the observed improvements, particularly the changes in social, emotional, cognitive, and academic self‐efficacy. However, an important distinction should be made between theoretical consistency and empirical demonstration. This study did not directly measure mastery experiences, vicarious experiences, verbal persuasion, physiological states, or affective states as mediating variables. Consequently, the study cannot establish that any of these mechanisms caused the observed changes. Social cognitive theory provides a plausible theoretical explanation for the pattern of findings, but these mechanisms require direct testing in future research.

The large improvement in academic self‐efficacy may also be considered within this framework. Students who experience successful practice, constructive feedback, and opportunities to observe effective responses may develop stronger perceptions of competence [13]. Nevertheless, because academic self‐efficacy was measured as an outcome rather than the sources of self‐efficacy as mediators, this study cannot determine which source contributed most strongly to the observed improvement. Future studies could incorporate measures of mastery experiences, peer modeling, perceived feedback, emotional states, and self‐regulated learning to test these pathways directly.

### 4.2. Implications of the Findings

The findings have implications for nursing education because they suggest that EI development can be incorporated into undergraduate curricula using a relatively brief structured format. The significant improvements across all five self‐efficacy dimensions indicate that EI training may have educational relevance beyond emotional awareness alone. In particular, the strong improvements in cognitive and academic self‐efficacy suggest that EI training warrants consideration as one component of broader strategies aimed at supporting students’ confidence in managing academic demands.

The brief nature of the intervention is also relevant to curriculum implementation. Nursing curricula are often densely structured, and lengthy additional programs may be difficult to accommodate. The present intervention consisted of eight 30‐min sessions, representing a total of four hours of structured training. This format may therefore be adaptable to existing seminars, student‐development activities, simulation sessions, or clinical preparation programs. However, feasibility should not be equated with demonstrated long‐term effectiveness. Future implementation studies should examine whether the program can be delivered consistently across different cohorts and institutions and whether similar effects are observed under routine educational conditions.

From a nursing management perspective, the findings may have implications for the preparation of graduates for emotionally demanding clinical environments. EI and self‐efficacy are relevant to interpersonal functioning, coping, confidence, and professional development [43]. However, because clinical performance, leadership behavior, resilience, and workforce outcomes were not directly measured in this study, claims that the intervention improves these outcomes would be premature. These findings support the potential relevance of EI training to professional preparation, while longitudinal studies are needed to determine whether improvements in EI and self‐efficacy translate into measurable changes in clinical practice and professional outcomes.

### 4.3. Strengths and Limitations

Several strengths should be considered when interpreting the findings. First, the use of a two‐group pretest–posttest design allowed baseline outcomes to be compared before the intervention and changes to be examined over time. The absence of statistically significant pretest differences between groups supports their baseline comparability. Second, the study included a contemporaneous control group, allowing the observed intervention group changes to be considered in relation to changes occurring in students who did not receive the EI program. Third, the study examined five distinct dimensions of self‐efficacy rather than relying solely on a global self‐efficacy score, providing a more detailed picture of the outcomes associated with the intervention. Fourth, the intervention was structured and standardized, which may facilitate replication in other nursing education settings. Finally, the study provides evidence from Saudi Arabia, contributing to the relatively limited intervention literature on EI development among nursing students in this context.

Several limitations should nevertheless be acknowledged. First, the study used a quasi‐experimental rather than individually randomized design. Students were drawn from pre‐existing educational groups, and although baseline comparability was demonstrated, unmeasured differences between groups cannot be completely excluded. Accordingly, the findings should be interpreted as evidence of an association between participation in the EI training and improvement in the measured outcomes rather than definitive proof of causality.

Second, the study relied on self‐report measures. Although established instruments were used, self‐report responses may be affected by social desirability, response tendencies, and participants’ awareness of receiving the intervention. Future studies should combine self‐report measures with objective or multimethod outcomes, such as faculty ratings, behavioral observations, simulation‐based assessments, or performance measures [44, 45].

Third, the study assessed outcomes immediately following completion of the intervention and did not include a delayed follow‐up assessment. Therefore, the sustainability of the observed improvements cannot be determined. These results demonstrate immediate postintervention changes, but they do not establish whether these gains would persist, diminish, or increase over subsequent months. Future studies should include medium‐ and long‐term follow‐up assessments to examine the durability of EI and self‐efficacy improvements and determine whether they translate into longer‐term academic and professional outcomes.

Fourth, the study was conducted at a single public College of Nursing in Saudi Arabia. Institutional, educational, and cultural characteristics may influence intervention implementation and participants’ responses. Consequently, generalization to nursing students in other institutions, regions, or countries should be undertaken cautiously. Multicenter and cross‐cultural studies would strengthen the external validity of the findings.

Finally, because participants were aware of the intervention, the Hawthorne effect cannot be completely excluded. The additional attention associated with participation may have contributed to some of the observed improvement [46]. The presence of a control group reduces, but does not eliminate, this possibility [47]. Future studies could incorporate attention‐matched control conditions to distinguish the effects of EI‐specific content from the effects of receiving additional educational attention. Overall, these limitations indicate that the findings should be interpreted as evidence of immediate improvements associated with a structured EI intervention, rather than evidence of definitive long‐term effectiveness. Nevertheless, the consistency of the findings across EI and all five self‐efficacy dimensions, together with baseline comparability and the absence of comparable changes across most outcomes in the control group, supports further investigation of structured EI training in undergraduate nursing education.

### 4.4. Future Research

Future research should build on these findings using randomized controlled and multicenter designs with larger and more diverse samples. Importantly, subsequent studies should include delayed follow‐up assessments to determine whether improvements in EI and self‐efficacy are maintained over time. Research should also examine whether changes in EI and self‐efficacy translate into observable improvements in clinical communication, simulation performance, clinical competence, academic achievement, resilience, leadership development, and transition to professional practice.

Future studies should also investigate the mechanisms underlying the observed improvements. For example, measures of emotional regulation, self‐regulated learning, mastery experiences, peer modeling, perceived feedback, and affective states could be incorporated to determine whether these variables mediate the relationship between EI training and self‐efficacy. Such research would allow the theoretical interpretation based on social cognitive theory to be tested empirically rather than inferred from the intervention content.

Comparative studies examining different intervention durations, delivery formats, and educational strategies would also help determine whether longer programs produce greater or more sustained effects than brief interventions. In addition, implementation research should assess the feasibility, acceptability, fidelity, and cost of integrating EI training into existing nursing curricula. Such evidence would be particularly valuable for determining how EI programs can be scaled across nursing education institutions.

## 5. Conclusion

This study provides evidence suggesting that participation in a structured EI training program is associated with higher EI and multidimensional self‐efficacy among undergraduate nursing students. Using a quasi‐experimental two‐group pretest–posttest study design, students who participated in the intervention demonstrated meaningful improvements across emotional, social, cognitive, insistence, and academic domains of self‐efficacy, alongside overall gains in EI. These findings underscore the capacity of targeted educational interventions to strengthen emotional and psychological competencies essential for success in nursing education and practice [25].

The results highlight EI as a foundational component of nursing preparation rather than a peripheral soft skill. The findings suggest that participation in EI training may be associated with greater academic confidence, emotional resilience, and readiness for the interpersonal demands of clinical practice. Importantly, the intervention was brief, structured, and feasible to implement within an academic setting, suggesting its practicality for integration into existing nursing curricula [48].

Beyond its educational benefits, the intervention may have implications for nursing workforce development by strengthening competencies associated with leadership readiness, professional resilience, effective interpersonal communication, and self‐confidence. These attributes are increasingly recognized as essential for supporting a sustainable and high‐performing nursing workforce.

Future longitudinal studies are needed to determine whether the observed improvements in EI and multidimensional self‐efficacy are sustained beyond the immediate postintervention period and contribute to long‐term academic and professional outcomes. While further research is warranted to examine long‐term outcomes and broader applicability, the findings support the intentional inclusion of EI development within undergraduate nursing education. Integrating EI‐focused training may help support the development of emotionally competent and resilient nursing graduates, although further research is needed to confirm its long‐term effects on professional practice and workforce outcomes.

The study extends existing EI intervention research by demonstrating that a brief and feasible educational intervention may influence multiple dimensions of self‐efficacy beyond emotional functioning alone, particularly cognitive and academic self‐efficacy domains that are directly relevant to learning and professional development in nursing education [49].

**Nomenclature**
EI: Emotional intelligence

## Author Contributions

Conceptualization, Shaherah Yousef Andargeery and Enas Mohammed Lotfy; methodology, Shaherah Yousef Andargeery and Enas Mohammed Lotfy; software, Shaherah Yousef Andargeery; validation, Shaherah Yousef Andargeery and Enas Mohammed Lotfy; formal analysis, Shaherah Yousef Andargeery; investigation, Enas Mohammed Lotfy; resources, Shaherah Yousef Andargeery and Enas Mohammed Lotfy; data curation, Shaherah Yousef Andargeery; writing–original draft preparation, Shaherah Yousef Andargeery and Enas Mohammed Lotfy; writing–review and editing, Shaherah Yousef Andargeery; visualization, Shaherah Yousef Andargeery; supervision, Shaherah Yousef Andargeery and Enas Mohammed Lotfy; project administration, Enas Mohammed Lotfy; and funding acquisition, Shaherah Yousef Andargeery.

## Funding

This research was funded by Princess Nourah bint Abdulrahman University Researchers Supporting Project Number (PNURSP2026R447), Princess Nourah bint Abdulrahman University, Riyadh, Saudi Arabia. Also, a grant no. (NBU‐FPEJ‐2026‐3731‐01) was received from the Deanship of Scientific Research at Northern Border University, Arar, Saudi Arabia.

## Disclosure

All authors have read and agreed to the published version of the manuscript.

## Ethics Statement

The IRB at Princess Nourah bint Abdulrahman University, Riyadh, Saudi Arabia, approved the study. IRB approval No. 23‐1043; IRB Registration No. HAP‐01‐R‐059.

## Consent

Informed consent was obtained from the study participants. There is no identifiable information on the participants reported within the manuscript.

## Conflicts of Interest

The authors declare no conflicts of interest.

## Supporting Information

Additional supporting information can be found online in the Supporting Information section.

## Supporting information


**Supporting Information** CONSORT 2025 checklist of information to include when reporting a randomised trial∗

## Data Availability

The datasets generated and/or analyzed during this study are not publicly available due to data privacy but are available from the corresponding author upon reasonable request.

## References

[1] Hurley J. , Hutchinson M. , Kozlowski D. , Gadd M. , and Vorst S. V. , Emotional Intelligence as a Mechanism to Build Resilience and Non‐Technical Skills in Undergraduate Nurses Undertaking Clinical Placement, International Journal of Mental Health Nursing. (2019) 29, no. 1, 47–55, 10.1111/inm.12607. 31127972

[2] Dugué M. , Sirost O. , and Dosseville F. , A Literature Review of Emotional Intelligence and Nursing Education, Nurse Education in Practice. (2021) 54, 10.1016/j.nepr.2021.103124. 34175653

[3] Cuartero N. and Tur A. , Emotional Intelligence, Resilience and Personality Traits Neuroticism and Extraversion: Predictive Capacity in Perceived Academic Efficacy, Nurse Education Today. (2021) 102, 10.1016/j.nedt.2021.104933. 33957394

[4] Dogham R. S. , Elcokany N. M. , Ghaly A. S. , El‐Ashry A. M. , and Ali H. F. M. , Emotional Intelligence and Reflective Thinking: A Synergistic Approach in Nursing Education, BMC Nursing. (2025) 24, no. 1, 10.1186/s12912-025-03175-w. PMC12082956 40380149

[5] Gamage C. K. W. and Sudusinghe W. S. , Unveiling the Heart of Nursing: The Transformative Impact of Emotional Intelligence on Patient-Centred Care, Asian Journal of Internal Medicine. (2024) 3, no. 1, 36–41, 10.4038/ajim.v3i1.129.

[6] Eranhikkal F. and Ahirwar G. , Understanding Emotional Intelligence, Coping Strategies, and Demographics Among Healthcare Professionals: Implications for Well-Being and Resilience, International Journal of Science and Research. (2025) 1051–1059, 10.21275/sr251017152035.

[7] Faria N. , Ramalhal T. , and Lucas P. , Scoping Review: The Emotional Intelligence of Nurses in the Clinical Care Environment, Annals of Medicine. (2019) 51, no. 1, 10.1080/07853890.2018.1560166.

[8] Dugué M. , Sirost O. , Heutte N. , and Dosseville F. , Emotional Intelligence Training in Health Students: A Pilot Study, Frontiers in Sports and Active Living. (2025) 7, 10.3389/fspor.2025.1511851. PMC11891197 40066062

[9] Schoeps K. , Barrera U. de la , and Montoya‐Castilla I. , Impact of Emotional Development Intervention Program on Subjective Well-Being of University Students, Higher Education. (2019) 79, no. 4, 711–729, 10.1007/s10734-019-00433-0.

[10] Shablizi M. S. , Yakhchali A. H. , Yailagh M. S. , and Maktabi G. , The Effect of Emotional Intelligence Training on Academic Commitment, Emotional Self-Regulation, and Academic Performance of Male Students at Farhangian University in Khuzestan Province, Health Nexus. (2025) 3, no. 2, 26–33, 10.61838/kman.hn.3.2.4.

[11] Dugué M. , Dosseville F. , Lavaud V. , Thomasse E. , and Sirost O. , Compétences Émotionnelles Et Simulation, Revue Education Santé Sociétés. (2024) 1–18, 10.17184/eac.8131.

[12] Riaz M. , Roger R. , Bashir S. , Javed K. , Charles R. , and Khursheed U. , Communication Skills and Emotional Intelligence Among Nursing Students on Patient’s Outcome, Nursearcher. (2025) Journal of Nursing & Midwifery Sciences, 67–72, 10.54393/nrs.v5i1.148.

[13] Bandura A. , Self-Efficacy: Toward a Unifying Theory of Behavioral Change, Psychological Review. (1977) 84, no. 2, 191–215, 10.1037/0033-295x.84.2.191. 847061

[14] Gómara A. O. , Miguél M. S. de , Elguea J. O. de , and Elguea A. O. de. , Testing General Self‐Efficacy, Perceived Competence, Resilience, and Stress Among Nursing Students: An Integrator Evaluation, Nursing and Health Sciences. (2020) 22, no. 3, 529–538, 10.1111/nhs.12689. 32128979

[15] Kiftiyah K. , Farida I. A. , Wiyono B. B. , and Hitipeuw I. , The Role Resilience And Academic Self-Efficacy In Increasing Academic Engagement Of Undergraduate Nursing Students, International Journal of Nursing and Midwifery Science (IJNMS). (2025) 8, no. 3, 363–374, 10.29082/ijnms/2024/vol8/iss3/630.

[16] Michinov E. , Robin G. , Hémon B. , Béranger R. , and Boissart M. , Protective Resources Against Stress Among Student Nurses: Influences of Self-Efficacy, Emotional Intelligence and Conflict Management Styles, HAL (Le Centre Pour La Communication Scientifique Directe). (2023) 74, 10.1016/j.nepr.2023.103849. 38006646

[17] Aldhahi M. I. , Alqahtani A. S. , Baattaiah B. A. , and Almohammed H. I. , Exploring the Relationship Between Students’ Learning Satisfaction and Self-Efficacy During the Emergency Transition to Remote Learning amid the Coronavirus Pandemic: A Cross-Sectional Study, Education and Information Technologies. (2021) 27, no. 1, 1323–1340, 10.1007/s10639-021-10644-7. PMC8275635 34276239

[18] Prifti R. , Self-Efficacy and Student Satisfaction in the Context of Blended Learning Courses, Open Learning The Journal of Open Distance and E-Learning. (2020) 37, no. 2, 111–125, 10.1080/02680513.2020.1755642.

[19] Shubayr N. and Dailah H. G. , Assessment of Emotional Intelligence, Self-Efficacy, and Perceived Stress Among Nursing Students in Clinical Practice: A Cross-Sectional Study, BMC Nursing. (2025) 24, no. 1, 10.1186/s12912-025-03109-6. PMC12065299 40346588

[20] Chang Y. and Tsai Y. , The Effect of University Students’ Emotional Intelligence, Learning Motivation and Self-Efficacy on Their Academic Achievement-Online English Courses, Frontiers in Psychology. (2022) 13, 10.3389/fpsyg.2022.818929. PMC8888520 35250754

[21] Tang Y. and He W. , Relationship Between Emotional Intelligence and Learning Motivation Among College Students During the COVID-19 Pandemic: A Serial Mediation Model, Frontiers in Psychology. (2023) 14, 10.3389/fpsyg.2023.1109569. PMC10050401 37008860

[22] Leal L. A. , Cassiano C. , Cruchinho P. et al., Strategical Pedagogy for the Development of Socio-Emotional Competences in Nursing Students, Nursing Reports. (2024) 14, no. 4, 2837–2849, 10.3390/nursrep14040208. PMC11503430 39449445

[23] Matroud F. , Othman M. S. , Jamil M. R. M. et al., Emotional Intelligence Trends in Nursing Education: Review the Literature, International Journal of Public Health Science. (2024) 14, no. 1, 10.11591/ijphs.v14i1.24584.

[24] Sayson J. D. V. , Navigating Unprecedented Challenges: A Narrative Review of Socio-Emotional Learning Competencies and Emotional Intelligence Among Nurses and Nursing Students in the COVID-19 Era, International Journal of Research and Review. (2023) 10, no. 12, 672–692, 10.52403/ijrr.20231268.

[25] Silva T. M. H. R. da. , Fostering Emotional Intelligence for Competency Development in Nursing Education, Advances in Educational Technologies and Instructional Design Book Series, 2024, IGI Global, 39–62, 10.4018/979-8-3693-7555-6.ch002.

[26] Jiménez‐Rodríguez D. , Jurado M. , del M. M. et al., The Effects of a Non-Technical Skills Training Program on Emotional Intelligence and Resilience in Undergraduate Nursing Students, Healthcare. (2022) 10, no. 5, 10.3390/healthcare10050866. PMC9141638 35628003

[27] Benjamin L. S. , an E. P. , Vijayalakshmi K. et al., Emotional Intelligence and Self-Esteem Among Saudi Arabian and Indian Nursing Students: Findings From Two Countries, BMC Nursing. (2024) 23, no. 1, 10.1186/s12912-024-02022-8. PMC11127298 38790031

[28] Hamad M. and Gurbutt R. , Emotional Intelligence in Preregistration Nurse Education, Nursing Standard. (2023) 38, no. 11, 61–66, 10.7748/ns.2023.e11994. 37731299

[29] Schutte N. S. , Malouff J. M. , Hall L. et al., Development and Validation of a Measure of Emotional Intelligence, Personality and Individual Differences. (1998) 25, no. 2, 167–177, 10.1016/s0191-8869(98)00001-4.

[30] Salovey P. and Mayer J. D. , Emotional Intelligence, Imagination, Cognition and Personality. (1990) 9, no. 3, 185–211, 10.2190/dugg-p24e-52wk-6cdg.

[31] O’Connor P. , Hill A. , Kaya M. , and Martin B. , The Measurement of Emotional Intelligence: A Critical Review of the Literature and Recommendations for Researchers and Practitioners, Frontiers in Psychology. (2019) 10, 10.3389/fpsyg.2019.01116. PMC6546921 31191383

[32] Ayed A. , Aqtam I. , Malak M. Z. et al., Insights into the Relationship Between Emotional Intelligence and Critical Thinking Among Nursing Students, BMC Nursing. (2025) 24, no. 1, 10.1186/s12912-025-03782-7. PMC12374380 40849675

[33] Alwan S. T. , Perceived self-efficacy Among Students in Baghdad University, Educational and Psychological Research Journal. (2013) 245–248.

[34] Ta’an W. , Rababah J. A. , Al‐Hammouri M. M. , Yousef J. , Mukattash T. L. , and Williams B. , Validation and Cross-Cultural Adaptation of the Six-Dimension Scale of Nursing Performance-Arabic Version, BMC Nursing. (2024) 23, no. 1, 10.1186/s12912-024-01740-3. PMC10797931 38238753

[35] Corbí R. G. , Pozo-Rico T. , Sánchez B. S. , and Costa J. L. C. , Can Emotional Intelligence be Improved? A Randomized Experimental Study of a Business-Oriented EI Training Program for Senior Managers, PLoS One. (2019) 14, no. 10, 10.1371/journal.pone.0224254. PMC6808549 31644585

[36] Horowitz E. , Kaminsky A. , Sidhu M. S. et al., Feasibility and Impact of Emotional Intelligence Training in Preclinical Medical Education, BMC Medical Education. (2026) 26, no. 1, 10.1186/s12909-026-08581-5. PMC12874979 41535874

[37] Yüceyurt N. K. and Kaya H. , Program Development Study Focusing on Emotional Intelligence Skills of Nursing Students: Randomized Controlled Trial, BMC Nursing. (2025) 24, no. 1, 10.1186/s12912-025-03491-1. PMC12232706 40624641

[38] Puertas-Molero P. , Ortega F. Z. , Chacon-Cuberos R. , Sánchez M. C. , Ramirez-Granizo I. , and Valero G. G. , La Inteligencia Emocional En El Ámbito Educativo: Un Meta-Análisis, Anales de Psicología. (2019) 36, no. 1, 84–91, 10.6018/analesps.345901.

[39] Durham M. , Smith R. , Cloonan S. et al., Development and Validation of an Online Emotional Intelligence Training Program, Frontiers in Psychology. (2023) 14, 10.3389/fpsyg.2023.1221817. PMC10470876 37663347

[40] MacCann C. , Jiang Y. , Brown L. E. R. , Double K. S. , Bucich M. , and Minbashian A. , Emotional Intelligence Predicts Academic Performance: A Meta-Analysis, Psychological Bulletin. (2019) 146, no. 2, 150–186, 10.1037/bul0000219. 31829667

[41] Shengyao Y. , Samsudin N. , Jenatabadi H. S. , and Xuefen L. , Emotional Intelligence and Academic Resilience: The Mediating Role of Cognitive-Motivational Factors, Humanities and Social Sciences Communications. (2026) 13, no. 1, 10.1057/s41599-026-07822-8.

[42] Taylor M. , Hoch J. M. , and Porter K. , Educational Interventions to Improve Emotional Intelligence in Nursing and Medical Students: A Systematic Review, Journal of Nursing Education and Practice. (2022) 13, no. 1, 10.5430/jnep.v13n1p19.

[43] Ludwika W. and Zuzanna B. , Samoregulacja Emocji a Poczucie Własnej Skuteczności Pielęgniarek W Opiece Nad Innymi=Emotional Self-Regulation and Self-Efficacy Among Nurses in the Context of Caring for Others, Digital Library of Zielona gora (University of Zielona Góra), 2025, University of Zielona Góra, https://zbc.uz.zgora.pl/dlibra/publication/103175/edition/88483/content.

[44] Brown A. W. , Altman D. G. , Baranowski T. et al., Childhood Obesity Intervention Studies: a Narrative Review and Guide for Investigators, Authors, Editors, Reviewers, Journalists, and Readers to Guard Against Exaggerated Effectiveness Claims, Obesity Reviews. (2019) 20, no. 11, 1523–1541, 10.1111/obr.12923. PMC7436851 31426126

[45] Soland J. and Gilbert J. , Does Socially Desirable Responding Change After an Intervention? Implications for Estimating Treatment Effects, 2026, 10.31234/osf.io/ujx4n_v3.

[46] Song M. and Ward S. E. , Assessment Effects in Educational and Psychosocial Intervention Trials: An Important but Often-Overlooked Problem: Assessment Effects in Intervention Trials, UNC Libraries. (2020) 10.17615/fqxz-dj60. PMC4439294 25728502

[47] Stevens J. , Mills S. D. , Millett T. J. , Lin F. , and Leeman J. , Design of a Dual Randomized Trial in a Type 2 Hybrid Effectiveness-Implementation Study, Implementation Science. (2023) 18, no. 1, 10.1186/s13012-023-01317-9. PMC10666326 37996884

[48] Fowler K. R. , Robbins L. K. , Field A. , and Avitia G. , Role of Emotional Intelligence in Nursing Leadership, Nursing Management. (2025) 56, no. 5, 19–25, 10.1097/nmg.0000000000000251. 40366852

[49] Sudrajat D. A. , Mailani F. , Ibrahim M. , and Kumala T. F. , The Effect of Emotional Intelligence Training on Self-Efficacy in Nursing Students, KnE Life Sciences. (2022) 10.18502/kls.v7i2.10328.

